# Contributions of Service-Learning on PETE Students’ Effective Personality: A Mixed Methods Research

**DOI:** 10.3390/ijerph17238756

**Published:** 2020-11-25

**Authors:** Oscar Chiva-Bartoll, Antonio Baena-Extremera, David Hortiguela-Alcalá, Pedro Jesús Ruiz-Montero

**Affiliations:** 1 Department of Education and Specific Didactics, Faculty of Humanities and Social Sciences, Universitat Jaume I, 12071 Castellón, Spain; ochiva@uji.es; 2 Department of Education Sciences, Faculty of Education, University Campus of Cartuja, University of Granada, s/n, 18071 Granada, Spain; abaenaextrem@ugr.es; 3 Department of Specific Didactics, Faculty of Education, University of Burgos, 09001 Burgos, Spain; dhortiguela@ubu.es; 4 Department of Physical Education and Sport, Faculty of Education and Sport Sciences, Campus of Melilla, University of Granada, 52071 Melilla, Spain

**Keywords:** service-learning, pedagogical model, physical education, effective personality, mixed methods, teacher training

## Abstract

Service-learning (SL) is a pedagogical model focused on achieving curricular goals while providing a community service. Previous research suggests that SL might promote qualities such as self-esteem, motivation, problem-focused coping, decision-making, empathy, and communication, which are associated with a psychological construct known as students’ Effective Personality (EP). These studies, however, did not specifically analyse the direct effects of SL on this construct. The aim of this study is to explicitly analyse the effect of SL on Physical Education Teacher Education (PETE) students’ EP using a mixed methods approach. The quantitative part of the approach followed a quasi-experimental design using the validated “Effective Personality Questionnaire for University Students”, which includes four dimensions: “Academic self-efficacy”, “Social self-realisation”, “Self-esteem”, and “Resolutive self-efficacy”. A non-probabilistic sampling on a total of 181 PETE students was then carried out, with 98 participating in the experimental group (42 male, 56 female), and 83 in the control group (34 male, 49 female). The comparisons revealed significant improvements in the experimental group, especially in the social self-realisation and resolutive self-efficacy dimensions. These findings were complemented by a qualitative analysis of 12 students’ semi-structured interviews. In conclusion, the study reported a positive influence of SL on the PETE students’ EP, providing valuable design patterns for future SL implementations.

## 1. Introduction

Higher education must renew its pedagogical paradigm by placing students at the centre of the teaching-learning process. In order to promote effective training programs, many kinds of pedagogical innovations are emerging in higher education, some of which emphasise and provide experiential and participatory learning scenarios aligned with a firm ethical commitment [1]. One such innovation is service-learning (SL), a pedagogical model focused on achieving curricular and social learnings while providing a social service to the community. One of the most widespread and accepted definitions of SL was supplied by Bringle and Hatcher [2], who describe it as an educational experience in which students take part in structured community service activities that meet social needs while gaining further understanding of the curricular contents through organized reflection processes [3]. From a pedagogical point of view, SL combines experiential learning [4] and problem-based learning [5,6].

In SL interventions, the social and psychological domains acquire a prominent role since students must adapt themselves to achieve both curricular and social targets [7,8]. The growth of SL implementations over the last decade has increased research interest on the topic at all educational levels and academic fields [9,10,11]. In particular, research has shown that SL, as a form of active learning, is a high-impact educational practice at the higher education level [12].

Particularly, the SL model has been widely used in Physical Education Teacher Education (PETE) [13,14] since it offers important tools that support the personal and social development of PETE students. Initially, Billig [15] and Eyler and Giles [16] classified the results of the SL effects into four categories: academic outcomes, personal outcomes, social outcomes, and citizenship values. More recently, Furco [17] grouped the SL outcomes into six different categories, drawing upon several research evidence from recent contributions: (1) students’ academic and cognitive development [7,8,18], (2) students’ civic engagement [7,19,20], (3) students’ vocational and professional development [21], (4) students’ ethical and moral growth [22], (5) students’ personal and identity growth [23], and (6) students’ social development [24,25]. Therefore, one can sensibly hypothesise that SL can contribute to the development of Effective Personality (EP).

EP is a psychological construct that collects some of the most relevant aspects of personal and social skills. The construct methodically combines several theoretical-empirical advancements during the last few decades, including Sternberg’s triarchic theory [26], Gardner’s work on multiple intelligences [27], Mayer and Salovey’s work on emotional intelligence [28], Bandura’s theory on self-efficacy [29], Heath’s psychological maturity model [30], and Bar-On’s theories on emotional-social intelligence [31]. Specifically, Pizarro, Martín, and Cortés [32] noted that the EP construct is composed of a number of dimensions that can be grouped into four mutually interacting categories: (1) “personal strengths”, including self-concept and self-esteem; (2) “personal demands”, including motivation, attribution, and expectations; (3) “personal challenges”, including coping with problems and decision making; and (4) “personal relationships”, including communication, assertiveness, and empathy [33]. Likewise, EP is a construct whose personality characteristics are related to professional and/or academic scenarios such as those posed by the SL pedagogical model.

The causal relationship between SL and EP seems reasonable in higher education settings, since, as we have seen in the preceding paragraphs, the effects of SL analysed to date suggest that it might improve features of the EP construct. These studies, however, focused on different partial features of SL rather than its relationship with the construct. Therefore, it is necessary to address studies that analyse the direct effects of SL on EP. By focusing on this particular aspect of the research, which aligns with the higher education challenge of updating its pedagogical paradigm towards a more effective training, we hypothesize that SL could improve the PETE students’ EP. Particularly, this is supported by current literature that suggests students’ improvements in self-concept and self-esteem can be made through personal reflection about oneself, which in turn encourages students to adjust their behaviour within the class group, thus increasing their social competences and communication skills [20]. Likewise, the inherent SL reflection processes would allow students to increase their motivation towards the task and professional-related skills [8,34,35,36,37]. Similarly, successful social experiences might help students readjust their expectations, become more aware of their skills, and develop greater potential to cope with problems in order to achieve their social goals [7,24,38]. Finally, students’ empathy for others might increase when they interact not only with their classmates but also with the community members involved in the programs [20,39,40]. It is therefore reasonable to investigate EP development as a consequence of innovative educational programs in higher education like SL. The specific research goal of this study is to analyse the contributions of a SL program on PETE students’ EP.

## 2. Materials and Methods

### 2.1. Participants

To select the sample for the quantitative aspect of the study, a quasi-experimental design was developed from a non-probabilistic and convenience sampling of two different groups (experimental and control) that totalled 181 voluntary participants from the Jaume I University PETE degree. A total of 98 PETE students participated in the experimental group (42 male and 56 female; median age = 24.00, IQR = 1.00), and 83 students participated in the control group (34 male and 49 female; median age = 24.00, IQR = 0.25). Two SL editions of the same SL program were consecutively carried out in the 2016/17 and 2017/18 academic years. Apart from a few students that decided not to participate due to various personal reasons, the majority of the PETE students that completed one out of the two editions of the program were part of the experimental group.

For the qualitative portion of the study, an intentional sample of 12 PETE students was used [41]. The reason for this option of recruitment was to obtain interpretations of PETE students that were representative of the participants in the quantitative approach. The criteria were: (1) sex, (2) global grades, and (3) academic year when the SL was carried out. The sample comprised one representative informant for each feature (Table 1).

### 2.2. Research Design

There are a number of difficulties with quasi-experimental methodological designs for ecological studies of innovative pedagogical models in higher education. A genuine control is almost impossible, and problems with separating groups often result in the contamination of designs [42]. According to Kember’s point of view [42], educational studies are complex since there are many variables involved. As an alternative, the author recommends triangulation across mixed-method designs from different sources. Keeping these challenges in mind, this study used a mixed method with methodological triangulation with a predominantly quantitative explanatory design [43,44]. This methodological approach implies collecting and analysing quantitative and then qualitative data within one study, combining both but giving more importance to the former [45]. Indeed, the mixed methods approach is one of the most extended modalities in Western educational research [46] and is supported for SL studies [9,24].

**Figure 1:**
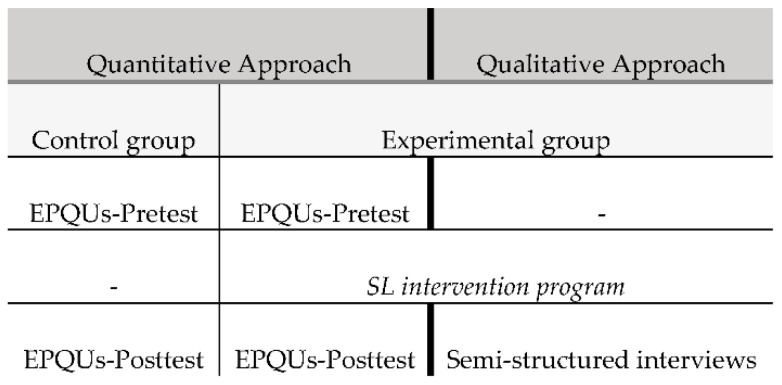
Mixed methods with predominantly quasi-experimental quantitative design used.

Following the mixed methods described, a quantitative quasi-experimental design was applied to carry out the quantitative approach, measuring the EP differences between an experimental group that experienced the SL intervention and a control group that did not. A qualitative approach was then carried out with 12 PETE students in order to complement the quantitative results by addressing their reflections and experiences [44,45]. The informants were chosen following a distribution that allowed the research team to obtain the perspective of different sexes and academic levels (Figure 1).

### 2.3. Intervention Program

While PETE students of the control group followed a traditional educational approach based on attending master-classes, practical sessions, and the successful completion of a theoretical essay and a final exam, the experimental group implemented an SL program whose purpose was to achieve the same curricular contents while facilitating social inclusion of children with special educational needs (SEN) through physical activity practise [47]. PETE students involved in the experimental group were expected to learn curricular contents by designing, teaching, and assessing the tasks provided to children with SEN. Both groups (traditional education and experimental methodology by SL) received training from the same team of two lecturers. In the formative planning of both groups the teacher educators scheduled tasks to ensure that all the students, regardless the group, spent approximately the same amount of time to the subject. Following Hastie’s [48] recommendations for describing pedagogical models in research publications (context, curricular elements, and implementation actions), the SL program consisted of:

Context: In each edition of the programme the intervention aimed to facilitate social inclusion through physical activities to the same 116 children (61 boys and 55 girls), ages 4 to 13 years old, with SEN caused by Down Syndrome (17 boys and 21 girls), Autism Spectrum Disorder (18 boys and 6 girls), Cerebral Palsy (9 boys and 5 girls), Attention-Deficit/Hyperactivity Disorder (17 boys and 22 girls), and Rett’s Syndrome (1 girl). The contact with these groups of children with SEN was made through social entities whose qualified staff had previously made the diagnoses. These children did not have an offer of extracurricular physical sport activities adjusted to their needs.

Curricular elements: Since SL is based on experiential learning [4], the curricular elements involved facilitated an educational praxis based on real problems, attempting to give significance and authenticity to the learning. The curricular objectives approached through the SL intervention were to

- Reflect on the teaching-learning processes and their social and personal implications.
- Adapt practices to functional diversity in physical education.
- Understand the principles that contribute to cultural, personal, and social education from physical education.
- Know, differentiate, and apply different teaching methods and styles according to the level of the students, the characteristics of the content, and the teachers’ own idiosyncrasies.
- Assess physical condition and recommend health-oriented physical exercises.
- Encourage and promote the practise of long-lasting and autonomous physical activities and sport habits among different populations.

Implementation actions: The program consisted of designing and leading physical activity sessions following guidelines related to organisational and educational approaches used in similar experiences [47]. Specifically, the PETE students were organized and worked with small groups at the rate of 2–3 PETE students for 5–7 children with SEN, distributed according to the ages, needs, and abilities of the children involved. PETE students accomplished 20 h of direct contact with the children with SEN, all participating equally in the different actions of the SL program. The SL program followed the Kolb’s [4] cycle of experiential learning, based on the following four stages:

- Concrete experience. PETE students carried out concrete experience actions in order to establish initial contact with the children with SEN. Through this concrete experience, PETE students could know and understand the children with SEN needs. This phase involved visiting and contacting social entities or bringing professional staff from social entities to class in order to create alliances and make students aware of the main physical and social needs of the children with SEN.
- Reflective observation. This phase required students to develop reflection tasks about the events they experienced, giving them enough time to exchange opinions with each other and contrast the compiled information in order to suggest the SL program’s goals. This reflection process remained in place throughout the remainder of the SL program, encouraging students to give meaning to the learning processes they acquired.
- Abstract conceptualisation. This third phase focused on students’ involvement with the curricular content. Once the needs to be faced were established, the project required a theoretical deepening of the curricular contents, joining the learning objectives with those of service. This phase allowed for the design of a specific intervention program based on sessions of inclusive motor games. In this sense, the learning and application of knowledge during the SL program was strongly tied to the subject.
- Active experimentation. This phase implied the execution of the tasks designed. PETE students were expected to focus on acquiring curricular learnings and the social values associated to them. In this experimentation phase, improvements and variants were constantly proposed and assessed. Each implementation session was used as a laboratory of experiences.

### 2.4. Instruments and Procedures

The Effective Personality Questionnaire for University Students (EPQUs) by Gómez [49] was administered to both groups (experimental and control) before and after the intervention to measure the effect of the SL program on the EP of the PETE students involved. It is a Likert-type scale where respondents specify their level of agreement or disagreement on a symmetric scale composed of 5 progressive levels, with 1 being “totally disagree” and 5 “totally agree”. When analysing the questionnaire in terms of predictive reliability and content validity, Gómez [49] obtained a reliability of 0.87 on the Alpha Consistency Index (Cronbach’s Alpha) and acceptable results in the analysis. In the context of this study, an initial first-order factor analysis and a subsequent second-order factor analysis were carried out in order to reduce the initial factors and simplify subsequent analyses. From this factorial analysis, which was executed as a multivariate analysis, it was concluded that the items of the resulting dimensions were maximally related to each other and minimally to those of other subsets. Finally, a third Confirmatory Factor Analysis (CFA) was carried out and framed in the Structural Equation Models (SEM), indicating that the data reasonably fit the proposed theoretical model. The questionnaire included 30 items distributed in four content dimensions: (1) “Academic self-efficacy”, defined from the expectations and attributions of academic performance in which students feel fulfilled (e.g., Item 5–My success in a subject is due to my dedication and good work); (2) “Social self-realisation”, understood as a link between the self-perception of the ability to establish and maintain social relationships and the expectations of the success of these interactions (e.g., Item 22–My successes in relationships with others are due to my ability to make friends); (3) “Self-esteem”, which integrates evaluative individual aspects including self-appreciation and self-knowledge, believing and valuing one’s own personal and social abilities, and identifying individual limitations (e.g., Item 15–I accept myself as I am, with my qualities, limitations, and defects); and (4) “Resolutive self-efficacy”, understood as effective coping with challenges including planning decision-making, learning to accommodate to the demands of the moment, and collecting as much information as possible in order to assess and solve practical situations (e.g., Item 12–To make a decision, I gather all the information I can find).

The semi-structured interviews occurred in person and were audio-recorded and transcribed verbatim. Before starting each interview, the researcher made a brief introduction to the interviewees, clarifying aspects such as the use of recording solely for research purposes, the non-obligation to answer all questions, and the possibility of stopping the recording at any time during the interview. Interviews lasted an average of 40 min. The interviewer prepared a number of topic areas and questions related to the objectives and the mixed-method design of the study, as seen in Table 2.

### 2.5. Data Analysis

#### 2.5.1. Quantitative Data Analysis

The normal distribution of data was analysed using the Kolmogorov–Smirnov test. Since data did not show a normal distribution, non-parametric tests were used. Mann–Whitney U tests were performed to compare the baseline level of samples from the experimental and control groups. The Wilcoxon test was carried out to compare the pretest–posttest global differences and for the different dimensions in both experimental and control groups. In the different analysis, *p*-value > 0.05 was considered as a reasonable cut-off for statistical significance. The effect size was calculated using Cohen’s d value. It can be interpreted as small (0.2 < d < 0.5), medium (0.5 < d < 0.8), or large (0.8 < d) [50]. All statistical analyses were performed using the Statistical Package for Social Science (IBM SPSS Statistics for Windows 27.0. Armonk, NY, USA).

#### 2.5.2. Qualitative Data Analysis

The qualitative analysis consisted of a multiphase approach based on an initial open-coding phase and a second axial coding phase, assisted by the computer program NVivo_v10. After transcribing the interviews, the researchers first assigned initial codes to meaningful expressions or paragraphs, going back and forth through the data. Next, an axial coding process was carried out that focused on identifying content related to the EP questionnaire dimensions. In this phase, the researchers used the previously coded information, establishing categories related to the different dimensions since they were all theoretically saturated. To ensure the trustworthiness of this part of the study, several procedures were followed [51], including triangulation among different members of the research team and a member-checking process to guarantee that researchers accurately interpreted what participants meant.

### 2.6. Ethical Considerations

To ensure fidelity and responsible investigation, the study followed the ethical considerations established by the ethics committee of the research team’s university. In other words, the research followed the American Psychological Association’s [52] Ethical Principles of Psychologists and Code of Conduct. Before participating in the study, each student was informed of its purpose. Furthermore, written informed consent was obtained from all participants.

## 3. Results

### 3.1. Quantitative Findings

The Mann–Whitney U tests reported no statistically significant differences between the baseline levels of both groups before the SL intervention, so it was assumed that all the participants started with comparable levels. After the SL intervention, pretest–posttest analyses were carried out using Wilcoxon tests on both groups. While the control group did not show statistically significant differences, the experimental group did, obtaining a small effect size (Z = −2,24; *p* = 0.023; d = 0.317). When comparing the pretest–posttest results by dimensions (Table 3), the experimental group reported statistically significant differences in the “Social self-realisation” and “Resolutive self-efficacy” dimensions, whereas the control group did not report statistically significant differences in any dimension. In conclusion, the effect sizes obtained in the experimental group comparisons were small, except for the “Social self-realisation” dimension, which was of medium size.

Finally, although only two dimensions showed statistically significant differences, it may be interesting to highlight that all the mean values of the experimental group were slightly higher after the SL intervention, in accordance with the global pretest–posttest comparison.

### 3.2. Qualitative Findings

In order to understand the experiences of the PETE students regarding the SL intervention, this section reflects the qualitative findings obtained from analysing the interviews. To complement the quantitative results interpretation, the qualitative findings and categories were classified according to the dimensions of the EPQUs. This way, the qualitative analysis focused on identifying content related to the dimensions to develop a qualitative comprehension of the experience [43,44]. In this sense, the “Academic self-efficacy”, “Social self-realisation”, “Self-esteem”, and “Resolutive self-efficacy” dimensions were established as categories since they were all theoretically saturated. The following codes were used to protect the interviewees’ identities. Semi-structured interviews were identified by the acronym SI and the number of the PETE student that was assigned (1–12).

#### 3.2.1. Academic Self-Efficacy

Academic self-efficacy is a dimension that focuses on personal features such as motivation, expectations, and performance of academic responsibilities. Regarding this category, most students reported related comments and reflections. Because SL was an innovative pedagogical approach many of the students were not familiar with, it seems that some of them did not know how to act in the first stages of the implementation. Over time, however, it seems that, to a large extent, they felt that expectations were met. Indeed, there were numerous comments that endorse this idea:

At first, I didn’t really understand what this approach (SL) consisted of, but as the days went by I began to understand everything a little better. Finally, I understood that it all made sense and I even got to enjoy the experience (SI-10).

[D]uring the experience I didn’t see it clear. But in the end, after all the reflections and program assessment, I think we have met most of the proposed learning objectives (SI-1).

I think that we have been able to adapt ourselves very well and perform a good service. Now I would repeat the experience again (SI-3).

Other students, however, had less enthusiastic impressions about the program. Some interviewees confessed that they could not make sense of the academic experience for different reasons, as seen in SI-4′s response:

The goals of the program (SL) are worthy, I do not dispute that. But as a teaching-learning method I think it is very demanding. It is difficult to work with these children (children with SEN) because during our previous teacher training, we haven’t delved into their particular needs (SI-4).

In these particular cases, it seems that the students found too much distance between the requirements of the program (academic expectations) and their perceived abilities to accomplish it, as noted by SI-2 in their response:

We are mainly used to memorizing and delving deeply into theoretical contents. However, SL requires different skills such as the ability to organize ourselves, to apply what we have learned throughout our training, etc. I think there is a very big leap between what has been asked of us so far and the SL. (...) It has come to overwhelm me (SI-2).

In short, the qualitative analysis shows how the “Academic self-efficacy” is a dimension with a disparity of impressions. While most students perceived that they had sufficiently fulfilled their academic responsibilities, a few confessed to having felt overwhelmed, thus compromising their perception of academic self-efficacy.

#### 3.2.2. Social Self-Realisation

This dimension implies social skills such as communication, empathy, and assertiveness, among others. The analysis shows that there was a remarkable perception of social skill development among the students interviewed. The researchers expected this since SL promotes strong social interaction between the different participants involved (teachers, students, and recipients of the service). Particularly, this interaction seems to have improved certain communication abilities, as well as empathy and assertiveness. In relation to communication skills among students, SI-7 wrote:

To design the children’ physical activity sessions we had to hold a good number of previous meetings in which we learned to contrast different views and perspectives. At first it was not easy because everybody wanted to do what they thought that would work better, but in the end, we developed our own strategies to talk without arguing (…) For example, taking turns, discussing the advantages and disadvantages of the defended points of view…(SI-7).

Likewise, regarding interaction with the recipients of the service, SI-12 noted:

It was essential to be able to comment with the parents on the operation of the sessions. The first days it was hard for us to talk to them, it was like we were ashamed or something like that. But little by little we were strengthening relationships (SI-12).

Although empathy is a complex psychological construct to evaluate, there were many statements made that by the students that assert a perception of improvement, as demonstrated by SI-10 and SI-5:

[O]ne of the things I liked the most about the (SL) program was being able to put myself in the families’ shoes (SI-10).

Now I understand better the families of these children. It is clear that they live a constant struggle every day. It is a pity that society does not provide them more aid (SI-5).

Finally, the perception of acquiring assertiveness through the SL implementation is clearly referred and exemplified in a comment by SI-3:

We have really learned to work as a team and to share points of view, even if they are contradictory. (…) Sometimes you have to say what you think, because if you keep quiet and assume things that you don’t agree with, in the end it can be worse. (SI-3)

From the results obtained, it can be concluded that PETE students felt quite socially fulfilled, which could explain improvements in the social self-realisation dimension caused by the SL program.

#### 3.2.3. Self-Esteem

This dimension includes one’s ability to evaluate individual elements such as self-knowledge and self-appreciation, emphasising one’s own personal and social value while considering personal limitations as well. This category requires special attention since it is sustained upon many interviewees’ oppositional comments and mentions. In other words, while some students felt that SL helped them improve their self-esteem, a few others stated the opposite. We address the different perspectives below.

On the one hand, some interviewees confessed that regarding self-esteem the SL experience was not positive at all, as stated by SI-2:

I have no doubt that we have learned many valuable things. However, I don’t know if due to the continuous difficulties when applying the physical activity sessions, or due to the fact of seeing the children with SEN not achieving the settled objectives, I felt a little down (SI-2).

On the other hand, however, there were many interviewees who interpreted their participation in the SL program as a positive experience that made them feel good and increased their self-esteem, while maintaining an awareness of their limitations:

Most of the time I felt good during the program. I know many sessions could have been better, but I think we’ve done a great job with the children with SEN. It is clear that at the beginning most of the planned tasks didn’t go according to plan, but thanks to the after-class reflections we learnt how to do it better (SI-7).

We also observed some PETE students who seemed to have experienced both phases during the SL experience:

Knowing the reality of these children was hard. But one day a mother told me that her son was very motivated on the program. That made me understand that, somehow, we were contributing to their well-being. From that day on my perspective changed and I started to feel better (SI-12).

The findings clearly suggest that students’ self-esteem is also a complicated psychological factor that could be conditioned by many aspects that cannot be directly controlled or directed by the SL experience. Thus, it is reasonable to accept that the experience was not intense or long enough to improve all the participants’ self-esteem or that it led to experiences that might have been perceived differently by the PETE students depending on their personality.

#### 3.2.4. Resolutive Self-Efficacy

This dimension focuses on decision-making skills and the ability to cope with real challenges and problems using personal resources. As expected, the SL experience kept the students engaged in a continuous process of decision-making and coping with problems related to the teacher training. Therefore, the findings suggest that the SL experience enhanced the PETE students’ perceived capacity to apply the curricular knowledge in real contexts, thus reinforcing decision-making processes and favouring their confidence when coping with real problem-solving challenges.

I believe that the SL program has helped me to connect the theory of the subject involved with its practical possibilities. (…) In this sense I have learnt that designing on paper physical activity sessions for children with SEN is not the same thing as having to apply them and overcome all the difficulties that arise in the real world (SI-8).

This link between theory and practice, as well as the processes of overcoming frustration and gaining confidence when coping with practical problems, are recurring issues in the vast majority of interviews, as demonstrated by SI-10′s response:

I’d say that one of the main learnings acquired have to do with the confidence to react to the unexpected situations. In this sense, I think that as future teachers we all have come out stronger. The SL experience taught us that in real class situations any solution we can think of is better than getting stuck. It wasn’t easy, but it was worth it (SI-10).

Indeed, most interviewees reinforced this point of view:

Being a teacher is not just a matter of planning good classes, but of knowing how to apply what is planned (SI-1).

At first, we didn’t know what to do when something didn’t go as planned, but now we are used to facing those problems (SI-12).

Overall, the findings grouped in this category suggest that SL fosters relationships between theory and practice in EP teacher training, exerting a clear influence on the confidence and perceived resolutive self-efficacy of students when they face challenges derived from the entrusted teaching tasks. As some the interviewees pointed out, however, it was not always easy.

## 4. Discussion

The objective of the present study was to know to what extent participation in SL could contribute to the development of PETE students’ EP. A mixed methodological approach was carried out, obtaining a series of results that lead us to suggest a favourable effect of SL on EP development, which aligns with previous investigations on SL that reported the approach’s positive effect on aspects such as self-efficacy, self-concept, and civic attitudes [1,11,53]. Additionally, these findings are in line with the work of Buchanan et al. [54], which referred to the positive contribution of SL on whose professional growth. Likewise, Bernadowsky et al. [55] also highlighted that teacher training increased students’ ability to solve social problems after a SL intervention. Therefore, it seems that the contribution of SL to the development of EP is clear in these areas.

On the one hand, the quantitative analysis of the EPQUs showed statistically significant differences in the pretest–posttest measures of the experimental group, although with a small effect size. On the other hand, the analysis of the interviews, which gave voice to the participants, revealed numerous allusions to features related to the dimensions that make up the questionnaire. These results are thus complementary [44,45]. To obtain a more detailed approach, however, separate analyses were performed in regards to the following dimensions: “Academic self-efficacy”, “Social self-realisation”, “Self-esteem”, and “Resolutive self-efficacy”. They will be discussed individually in the following paragraphs.

In the case of the “Academic self-efficacy” dimension, the quantitative analysis (pretest–posttest of the experimental group) did not reveal statistically significant differences, comprising a small difference of the mean value after the SL intervention. Regarding the qualitative analysis, the results indicated that most interviewees’ comments were compatible with the development of this dimension; any reservations held by the students were likely caused by the difference between their perceived capacities and the SL requirements. Therefore, it seems that the results obtained from the different methodological approaches were consistent but inconclusive to an extent. Regarding the motivation aspect included in this dimension, however, our findings are fully consistent with those reported by Billig et al. [56], who also reported better scores after the SL intervention but lacked statistically significant differences. In contrast, our findings are not as conclusive as those presented by Gallini and Moely [57], whose findings attributed SL with the ability to motivate participants and increase their academic expectations and responsibilities, or those by Moser and Rogers [58], who asserted the role of SL in increasing students’ willingness to learn and their capacity for effort and expectations of success.

Regarding the “Social self-realisation” dimension, the quantitative results showed statistically significant differences in the pretest–posttest comparison of the experimental group, with a clear improvement in the mean scores supported by a medium effect size. These results were endorsed by the qualitative findings since the analysis of the interviews indicated PETE students’ positive perspectives. This aligns with various research studies that determined how SL experiences promote improvements in participating students’ social skills [7,20,59,60,61] and empathy [62]. Additionally, these social skills-related findings are consistent with some representative SL systematic reviews and meta-analyses [10,11,63]. It is important to keep in mind, however, that the traditionally analysed social skills are not necessarily the same as the social self-realisation dimension examined here. Therefore, this study provides an important and specific research finding regarding social skills since the social self-realisation analysed here refers to the students’ perceptions of their own social dimension rather than their capacities, abilities, or skills for interaction and socialisation.

In the “Self-esteem” dimension, no statistically significant differences were found. This result does not mean that SL did not affect a few PETE students, however, since the qualitative findings show a disparity in their interpretations. While some interviewees reported feelings of satisfaction due to the service they provided to the children with SEN and their families, others confessed that they felt a bit disappointed because they believed that they did not meet expectations [63]. All in all, this disparity in the results might be explained by the fact that self-esteem, despite being modifiable according to one’s own experiences, is not a personality trait susceptible to change in short periods of time.

In the “Resolutive self-efficacy” dimension, our quantitative findings revealed statistically significant differences in favour of the posttest measures of the experimental group, albeit with a small effect size. Our findings agree with several studies that point to the SL contribution on the improvement of skills such as decision making [1,64], and the personal and professional efficacy of students [55,65,66]. Furthermore, these quantitative results are complemented by the qualitative analysis, in which the PETE students that were interviewed expressed that the SL experience made them feel better equipped to apply the theoretical knowledge they previously acquired. These results align with findings reported in several studies that analysed SL interventions in teacher training settings [37,67,68]. Therefore, this study not only endorses the results of previous analyses but also offers a concrete perspective based on the construct of EP.

To a large extent, the results obtained meet the expectations underlying the study, providing a more specific view on the contributions of SL on students’ EP. However, although the findings reported are encouraging, some limitations must be considered. Firstly, the composition of the experimental and control groups by means of convenience recruitment must be taken into account; a randomized controlled sampling would have strengthened its validity [69]. In this vein, the teacher educators, who worked with both groups, were aware of the group assignment of the participants. However, this is a difficult issue to control in this type of ecological studies. Thirdly, the participants are not representative of any larger population, so results cannot be categorically generalised [70]. However, an attempt was made to counteract these issues by using a mixed methodological approach, allowing the researchers to triangulate different approaches and viewpoints. Finally, it is reasonable to warn that the outcomes and improvements might be interpreted as “cognitions”, “attitudes” and/or “skills” that one can acquire or develop, rather than consolidated traits.

## 5. Conclusions

The present study highlighted some of the contributions that SL participation had on PETE students’ EP. From the mixed methods approach carried out, the quantitative results showed that, despite small and medium effect sizes, the global EP improved significantly. Not all dimensions improved equally, however; when comparing dimensions, only two, “Social self-realisation” and “Resolutive self-efficacy”, obtained statistically significant differences. These results, in turn, are complemented and reinforced by the qualitative approach. The qualitative perspective indicated that “Social self-realisation” was the category most referred to in terms of empathy and the development of communication skills. The “Resolutive self-efficacy” dimension also obtained interesting results since the PETE students perceived the development of problem-solving skills. Interesting reflections were also obtained regarding “Self-esteem” and “Academic self-efficacy”, but perceived improvement was not reported in any of the cases. Although more research is needed in this field, this study found that teacher educators might consider SL implementation as an appropriate option for training effective PE teachers. In this sense, the study is valuable for future practitioners and teacher educators because tentative implications for the SL design patterns can be derived from the positive results obtained. Since the link between theory and practice seems to have been reinforced through this SL design, this study underlines the importance of the reflection phase in order to not only emotionally accompany the students, but to support them in applying curricular content during the active experimentation phase.

Regarding future research attempts, it would be interesting to compare different SL programs with variances in some aspects such as duration, intensity, type of service provided, etc. Similarly, it would be useful to more thoroughly investigate research that analysed the long-term effects of SL on both participant collectives involved, PETE students and receivers alike.

## Figures and Tables

**Table 1:** Characteristics of the Physical Education Teacher Education (PETE) students interviewed.

Global Grades	Male		Female	
	Academic Year		Academic Year	
	2016/17	2017/18	2016/17	2017/18
A	1	1	1	1
B	1	1	1	1
C+	1	1	1	1

**Table 2:** The general scheme of the interviews carried out.

Interview Parts	Issues	Basic Interview Guide
“Ice breaker” questions	Information on personal matters, educational experience, training attainments, and previous interactions of children with Special Educational Needs	-What is your academic experience? -Have you participated in similar educational experiences? -Have you been involved in service-learning programs before this one?
General questions	General impressions and feelings on the service-learning program experience.	-What is your general opinion regarding the service-learning experience? -What would you change from this educational experience? -How do you understand the relation between theory and practise in the Physical Education Teacher training?
Specific questions	Effective Personality-related questions.	-Would you highlight any specific learning or acquired skill from this experience? -What kind of personal and social skills do you perceive you have improved? -Do you feel more confident on your own capacities to solve improvised problems after this experience?
Conclusion question	Further observations (optional)	-Would you like to add any reflection that we have not previously discussed?

**Table 3:** Pretest–posttest group comparisons and effect sizes.

Dimension	Experimental Group (*n* = 98)				Control Group (*n* = 83)			
	Pretest (SD)	Posttest (SD)	*p*	*d*	Pretest (SD)	Posttest (SD)	*p*	*d*
Academic self-efficacy	3.773 (0.89)	3.796 (0.74)	0.378	0.187	3.701 (0.64)	3.724 (1.21)	0.478	0.123
Social self-realisation	3.681 (0.29)	4.267 (0.45)	0.003 *	0.512	3.698 (0.53)	3.871 (0.86)	0.116	0.172
Self-esteem	3.317 (1.12)	3.464 (0.97)	0.447	0.104	3.338 (1.01)	3.457 (1.22)	0.501	0.098
Resolutive self-efficacy	3.398 (0.56)	3.773 (0.33)	0.041 *	0.349	3.463 (0.67)	3.542 (0.83)	0.215	0.139

* *p* < 0.05.
